# Generalization in quantum machine learning from few training data

**DOI:** 10.1038/s41467-022-32550-3

**Published:** 2022-08-22

**Authors:** Matthias C. Caro, Hsin-Yuan Huang, M. Cerezo, Kunal Sharma, Andrew Sornborger, Lukasz Cincio, Patrick J. Coles

**Affiliations:** 1 Department of Mathematics, Technical University of Munich, Garching, Germany; 2 Munich Center for Quantum Science and Technology (MCQST), Munich, Germany; 3 Institute for Quantum Information and Matter, Caltech, Pasadena, CA USA; 4 Department of Computing and Mathematical Sciences, Caltech, Pasadena, CA USA; 5 Information Sciences, Los Alamos National Laboratory, Los Alamos, NM 87545 USA; 6 Center for Nonlinear Studies, Los Alamos National Laboratory, Los Alamos, NM 87545 USA; 7 Joint Center for Quantum Information and Computer Science, University of Maryland, College Park, MD 20742 USA; 8 Quantum Science Center, Oak Ridge, TN 37931 USA; 9 Theoretical Division, Los Alamos National Laboratory, Los Alamos, NM 87545 USA

**Keywords:** Quantum information, Computer science

## Abstract

Modern quantum machine learning (QML) methods involve variationally optimizing a parameterized quantum circuit on a training data set, and subsequently making predictions on a testing data set (i.e., generalizing). In this work, we provide a comprehensive study of generalization performance in QML after training on a limited number *N* of training data points. We show that the generalization error of a quantum machine learning model with *T* trainable gates scales at worst as $\sqrt{T/N}$. When only *K* ≪ *T* gates have undergone substantial change in the optimization process, we prove that the generalization error improves to $\sqrt{K/N}$. Our results imply that the compiling of unitaries into a polynomial number of native gates, a crucial application for the quantum computing industry that typically uses exponential-size training data, can be sped up significantly. We also show that classification of quantum states across a phase transition with a quantum convolutional neural network requires only a very small training data set. Other potential applications include learning quantum error correcting codes or quantum dynamical simulation. Our work injects new hope into the field of QML, as good generalization is guaranteed from few training data.

## Abstract (web summary)

The power of quantum machine learning algorithms based on parametrised quantum circuits are still not fully understood. Here, the authors report rigorous bounds on the generalisation error in variational QML, confirming how known implementable models generalize well from an efficient amount of training data.

## Introduction

The ultimate goal of machine learning (ML) is to make accurate predictions on unseen data. This is known as generalization, and significant effort has been expended to understand the generalization capabilities of classical ML models. For example, theoretical results have been formulated as upper bounds on the generalization error as a function of the training data size and the model complexity^1–5^. Such bounds provide guidance as to how much training data is required and/or sufficient to achieve accurate generalization.

Quantum machine learning (QML) is an emerging field that has generated great excitement^6–9^. Modern QML typically involves training a parameterized quantum circuit in order to analyze either classical or quantum data sets^10–16^. Early results indicate that, for classical data analysis, QML models may offer some advantage over classical models under certain circumstances^17–19^. It has also been proven that QML models can provide an exponential advantage in sample complexity for analyzing quantum data^20,21^.

However, little is known about the conditions needed for accurate generalization in QML. Significant progress has been made in understanding the trainability of QML models^18,22–36^, but trainability is a separate question from generalization^18,37,38^. Overfitting of training data could be an issue for QML, just as it is for classical machine learning. Moreover, the training data size required for QML generalization has yet to be fully studied. Naïvely, one could expect that an exponential number of training points are needed when training a function acting on an exponentially large Hilbert space. For instance, some studies have found that, exponentially in *n*, the number of qubits, large amounts of training data would be needed, assuming that one is trying to train an arbitrary unitary^39,40^. This is a concerning result, since it would imply exponential scaling of the resources required for QML, which is precisely what the field of quantum computation would like to avoid.

In practice, a more relevant scenario to consider instead of arbitrary unitaries is learning a unitary that can be represented by a polynomial-depth quantum circuit. This class of unitaries corresponds to those that can be efficiently implemented on a quantum computer, and it is exponentially smaller than that of arbitrary unitaries. More generally, one could consider a QML model with *T* parameterized gates and relate the training data size *N* needed for generalization to *T*. Even more general would be to consider generalization error a dynamic quantity that varies during the optimization.

In this work, we prove highly general theoretical bounds on the generalization error in variational QML: The generalization error is approximately upper bounded by $\sqrt{T/N}$. In our proofs, we first establish covering number bounds for the class of quantum operations that a variational QML model can implement. From these, we then derive generalization error bounds using the chaining technique for random processes. A key implication of our results is that an efficiently implementable QML model, one such that $T\in {{{{{{{\mathcal{O}}}}}}}}({{{{{{{\rm{poly}}}}}}}}n)$, only requires an efficient amount of training data, $N\in {{{{{{{\mathcal{O}}}}}}}}({{{{{{{\rm{poly}}}}}}}}n)$, to obtain good generalization. This implication, by itself, will improve the efficiency guarantees of variational quantum algorithms^10,41,42^ that employ training data, such as quantum autoencoders^13^, quantum generative adversarial networks^43^, variational quantum error correction^44,45^, variational quantum compiling^46,47^, and variational dynamical simulation^48–51^. It also yields improved efficiency guarantees for classical algorithms that simulate QML models.

We furthermore refine our bounds to account for the optimization process. We show that generalization improves if only some parameters have undergone substantial change during the optimization. Hence, even if we used a number of parameters *T* larger than the training data size *N*, the QML model could still generalize well if only some of the parameters have changed significantly. This suggests that QML researchers should be careful not to overtrain their models especially when the decrease in training error is insufficient.

To showcase our results, we consider quantum convolutional neural networks (QCNNs)^27,45^, a QML model that has received significant attention. QCNNs have only $T={{{{{{{\mathcal{O}}}}}}}}(\log n)$ parameters and yet they are capable of classifying quantum states into distinct phases. Our theory guarantees that QCNNs have good generalization error for quantum phase recognition with only polylogarithmic training resources, $N\in {{{{{{{\mathcal{O}}}}}}}}({\log }^{2}n)$. We support this guarantee with a numerical demonstration, which suggests that even constant-size training data can suffice.

Finally, we highlight the task of quantum compiling, a crucial application for the quantum computing industry. State-of-the-art classical methods for approximate optimal compiling of unitaries often employ exponentially large training data sets^52–54^. However, our work indicates that only polynomial-sized data sets are needed, suggesting that state-of-the-art compilers could be further improved. Indeed, we numerically demonstrate the surprisingly low data cost of compiling the quantum Fourier transform at relatively large scales.

## Results

### Framework

Let us first outline our theoretical framework. We consider a quantum machine learning model (QMLM) as being a parameterized quantum channel, i.e., a completely positive trace preserving (CPTP) map that is parameterized. We denote a QMLM as ${{{{{{{{\mathcal{E}}}}}}}}}_{{{{{{{{\boldsymbol{\alpha }}}}}}}}}^{{{{{{{{\rm{QMLM}}}}}}}}}(\cdot )$ where ***α*** = (***θ***, ***k***) denotes the set of parameters, including continuous parameters ***θ*** inside gates, as well as discrete parameters ***k*** that allow the gate structure to vary. We make no further assumptions on the form of the dependence of the CPTP map ${{{{{{{{\mathcal{E}}}}}}}}}_{{{{{{{{\boldsymbol{\alpha }}}}}}}}}^{{{{{{{{\rm{QMLM}}}}}}}}}(\cdot )$ on the parameters ***α***. During the training process, one would optimize the continuous parameters ***θ*** and potentially also the structure ***k*** of the QMLM.

A QMLM takes input data in the form of quantum states. For classical data *x*, the input is first encoded in a quantum state via a map *x* ↦ *ρ*(*x*). This allows the data to be either classical or quantum in nature, since regardless it is eventually encoded in a quantum state. We assume that the data encoding is fixed in advance and not optimized over. We remark here that our results also apply for more general encoding strategies involving data re-uploading^55^, as we explain in Supplementary Note 3.

For the sake of generality, we allow the QMLM to act on a subsystem of the state *ρ*(*x*). Hence, the output state can be written as $({{{{{{{{\mathcal{E}}}}}}}}}_{{{{{{{{\boldsymbol{\alpha }}}}}}}}}^{{{{{{{{\rm{QMLM}}}}}}}}}\otimes {{{{{{\mathrm{id}}}}}}})(\rho (x))$. For a given data point (*x*_*i*_, *y*_*i*_), we can write the loss function as

1 $$\ell ({{{{{{{\boldsymbol{\alpha }}}}}}}};{x}_{i},{y}_{i})={{{{{{{\rm{Tr}}}}}}}}\left[{O}_{{x}_{i},{y}_{i}}^{{{{{{{{\rm{loss}}}}}}}}}\left({{{{{{{{\mathcal{E}}}}}}}}}_{{{{{{{{\boldsymbol{\alpha }}}}}}}}}^{{{{{{{{\rm{QMLM}}}}}}}}}\otimes {{{{{{\mathrm{id}}}}}}}\right)(\rho ({x}_{i}))\right],$$

for some Hermitian observable ${O}_{{x}_{i},{y}_{i}}^{{{{{{{{\rm{loss}}}}}}}}}$. As is common in classical learning theory, the prediction error bounds will depend on the largest (absolute) value that the loss function can attain. In our case, we therefore assume ${C}_{{{{{{{{\rm{loss}}}}}}}}}:=\mathop{\sup }\nolimits_{x,y}||{O}_{x,y}^{{{{{{{{\rm{loss}}}}}}}}}|| < \infty$, i.e., the spectral norm can be bounded uniformly over all possible loss observables.

In Eq. (1), we take the measurement to act on a single copy of the output of the QMLM ${{{{{{{{\mathcal{E}}}}}}}}}_{{{{{{{{\boldsymbol{\alpha }}}}}}}}}^{{{{{{{{\rm{QMLM}}}}}}}}}(\cdot )$ upon input of (a subsystem of) the data encoding state *ρ*(*x*_*i*_). At first this looks like a restriction. However, note that one can choose ${{{{{{{{\mathcal{E}}}}}}}}}_{{{{{{{{\boldsymbol{\alpha }}}}}}}}}^{{{{{{{{\rm{QMLM}}}}}}}}}(\cdot )$ to be a tensor product of multiple copies of a QMLM, each with the same parameter setting, applied to multiple copies of the input state. Hence our framework is general enough to allow for global measurements on multiple copies. In this addition to the aforementioned situation, we further study the case in which trainable gates are more generally reused.

For a training dataset $S={\{({x}_{i},{y}_{i})\}}_{i=1}^{N}$ of size *N*, the average loss for parameters ***α*** on the training data is

2 $${\hat{R}}_{S}({{{{{{{\boldsymbol{\alpha }}}}}}}})=\frac{1}{N}\mathop{\sum }\limits_{i=1}^{N}\ell ({{{{{{{\boldsymbol{\alpha }}}}}}}};{x}_{i},{y}_{i}),$$

which is often referred to as the *training error*. When we obtain a new input *x*, the *prediction error* of a parameter setting ***α*** is taken to be the expected loss

3 $$R({{{{{{{\boldsymbol{\alpha }}}}}}}})=\mathop{{\mathbb{E}}}\limits_{(x,y) \sim P}\left[\ell ({{{{{{{\boldsymbol{\alpha }}}}}}}};x,y)\right],$$

where the expectation is with respect to the distribution *P* from which the training examples are generated.

Achieving small prediction error *R*(***α***) is the ultimate goal of (quantum) machine learning. As *P* is generally not known, the training error ${\hat{R}}_{S}({{{{{{{\boldsymbol{\alpha }}}}}}}})$ is often taken as a proxy for *R*(***α***). This strategy can be justified via bounds on the *generalization error*

4 $${{{{{{\mathrm{gen}}}}}}}\,({{{{{{{\boldsymbol{\alpha }}}}}}}})=R({{{{{{{\boldsymbol{\alpha }}}}}}}})-{\hat{R}}_{S}({{{{{{{\boldsymbol{\alpha }}}}}}}}),$$

which is the key quantity that we bound in our theorems.

### Analytical results

We prove probabilistic bounds on the generalization error of a QMLM. Our bounds guarantee that a good performance on a sufficiently large training data set implies, with high probability, a good performance on previously unseen data points. In particular, we provide a precise meaning of "sufficiently large” in terms of properties of the QMLM and the employed training procedure.

**Fig. 1:**
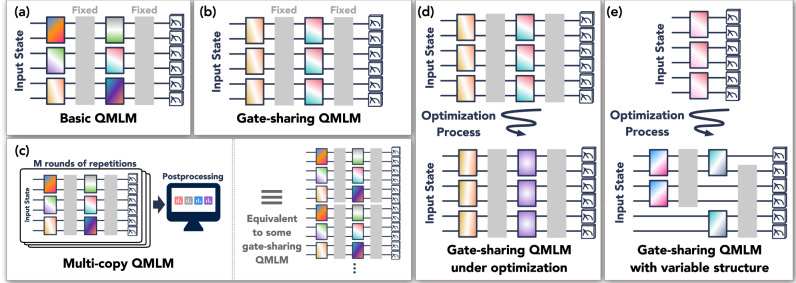
Various types of Quantum Machine Learning Models (QMLMs). Panel (**a**) depicts a basic QMLM with *T* = 6 independently parameterized gates. The gray boxes illustrate some global evolutions that are not trainable. Panel (**b**) shows a gate-sharing QMLM with *T* = 2 independently parameterized gates, each gate is repeatedly used for *M* = 3 times. In panel (**c**), we depict a multi-copy QMLM. We take measurement data from *M* rounds of a basic QMLM with *T* = 6 parameterized gates and post-process the measurement outcomes to produce an output. Running *M* copies of a basic QMLM with *T* gates is equivalent to running a gate-sharing QMLM with *T* = 6 parameterized gates, in which each gate is repeated *M* times. Panel (**d**) describes a gate-sharing QMLM under optimization. The parameterized gate to the left undergoes a small change, while the one to the right undergoes a large change. If we sort the changes Δ_1_, Δ_2_ from large to small, then Δ_1_ ≫ Δ_2_ ≈ 0. Finally, panel (**e**) illustrates gate-sharing QMLM with variable structure. The number *T* of parameterized gates changes throughout the optimization. The figure begins with *T* = 1 and ends with *T* = 2.

Figure 1 gives an overview of the different scenarios considered in this work. We begin with the basic form of our result. We consider a QMLM that has arbitrarily many non-trainable global quantum gates and *T* trainable local quantum gates. Here, by local we mean *κ*-local for some *n*-independent locality parameter *κ*, and a local quantum gate can be a unitary or a quantum channel acting on *κ* qubits. Then we have the following bound on the generalization error for the QMLM with final parameter setting α* after training:

#### Theorem 1

(*Basic QMLM*). For a QMLM with *T* parameterized local quantum channels, with high probability over training data of size *N*, we have that

5 $${{{{{{\mathrm{gen}}}}}}}\,({{{{{{{{\boldsymbol{\alpha }}}}}}}}}{*})\in {{{{{{{\mathcal{O}}}}}}}}\left(\sqrt{\frac{T\log T}{N}}\right).$$

#### Remark 1

Theorem 1 directly implies sample complexity bounds: For any *ε* > 0, we can, with high success probability, guarantee that gen(α*) ⩽ *ε*, already with training data of size $N \sim T\log T/{\varepsilon }^{2}$, which scales effectively linearly with *T*, the number of parameterized gates.

For efficiently implementable QMLMs with $T\in {{{{{{{\mathcal{O}}}}}}}}({{{{{{{\rm{poly}}}}}}}}n)$, a sample size of $N\in {{{{{{{\mathcal{O}}}}}}}}\left({{{{{{{\rm{poly}}}}}}}}n/{\varepsilon }^{2}\right)$ is already sufficient. More concretely, if $T\in {{{{{{{\mathcal{O}}}}}}}}\left({n}^{D}\right)$ for some degree *D*, then the corresponding sufficient sample complexity obtained from Theorem 1 satisfies $N\in \tilde{{{{{{{{\mathcal{O}}}}}}}}}\left({n}^{D}/{\varepsilon }^{2}\right)$, where the $\tilde{{{{{{{{\mathcal{O}}}}}}}}}$ hides factors logarithmic in *n*. In the NISQ era^56^, we expect the number *T* of trainable maps to only grow mildly with the number of qubits, e.g., as in the architectures discussed in refs. 18, 45, 57. In this case, Theorem 1 gives an especially strong guarantee.

In various QMLMs, such as QCNNs, the same parameterized local gates are applied repeatedly. One could also consider running the same QMLM multiple times to gather measurement data and then post-processing that data. In both cases, one should consider the QMLM as using the same parameterized local gates repeatedly. We assume each gate to be repeated at most *M* times. A direct application of Theorem 1 would suggest that we need a training data size *N* of roughly *MT*, the total number of parameterized gates. However, the required number of training data actually is much smaller:

#### Theorem 2

(*Gate-sharing QMLM*). Consider a QMLM with *T* independently parameterized local quantum channels, where each channel is reused at most *M* times. With high probability over training data of size *N*, we have

6 $${{{{{{\mathrm{gen}}}}}}}\,({{{{{{{{\boldsymbol{\alpha }}}}}}}}}{*})\in {{{{{{{\mathcal{O}}}}}}}}\left(\sqrt{\frac{T\log (MT)}{N}}\right).$$

Thus, good generalization, as in Remark 1, can already be guaranteed, with high probability, when the data size effectively scales linearly in *T* (the number of independently parameterized gates) and only logarithmically in *M* (the number of uses). In particular, applying multiple copies of the QMLM in parallel does not significantly worsen the generalization performance compared to a single copy. Thus, as we discuss in Supplementary Note 3, Theorem 2 ensures that we can increase the number of shots used to estimate expectation values at the QMLM output without substantially harming the generalization behavior.

The optimization process of the QMLM also plays an important role in the generalization performance. Suppose that during the optimization process, the *t*^th^ local gate changed by a distance Δ_*t*_. We can bound the generalization error by a function of the changes ${\{{{{\Delta }}}_{t}\}}_{t}$.

#### Theorem 3

(*Gate-sharing QMLM under optimization*). Consider a QMLM with *T* independently parameterized local quantum channels, where the *t*^th^ channel is reused at most *M* times and is changed by Δ_*t*_ during the optimization. Assume Δ_1_≥…≥Δ_*T*_. With high probability over training data of size *N*, we have

7 $${{{{{{\mathrm{gen}}}}}}}\,({{{{{{{{\boldsymbol{\alpha }}}}}}}}}{*})\in {{{{{{{\mathcal{O}}}}}}}}\left(\mathop{\min }\limits_{K=0,\ldots,T}\left\{\sqrt{\frac{K\log (MT)}{N}}+\mathop{\sum }\limits_{k=K+1}^{T}M{{{\Delta }}}_{k}\right\}\right).$$

When only *K* ≪ *T* local quantum gates have undergone a significant change, then the generalization error will scale at worst linearly with *K* and logarithmically in the total number of parameterized gates *MT*. Given that recent numerical results suggest that the parameters in a deep parameterized quantum circuit only change by a small amount during training^58,59^, Theorem 3 may find application in studying the generalization behavior of deep QMLMs.

Finally, we consider a more advanced type of variable ansatz optimization strategy that is also adopted in practice^60–63^. Instead of fixing the structure of the QMLM, such as the number of parameterized gates and how the parameterized gates are interleaved with the fixed gates, the optimization algorithm could vary the structure, e.g., by adding or deleting parameterized gates. We assume that for each number *T* of parameterized gates, there are *G*_*T*_ different QMLM architectures.

#### Theorem 4

(*Gate-sharing QMLM with variable structure*). Consider a QMLM with an arbitrary number of parameterized local quantum channels, where for each *T* > 0, we have *G*_*T*_ different QMLM architectures with *T* parameterized gates. Suppose that after optimizing on the data, the QMLM has *T* independently parameterized local quantum channels, each repeated at most *M* times. Then, with high probability over input training data of size *N*,

8 $${{{{{{\mathrm{gen}}}}}}}\,({{{{{{{{\boldsymbol{\alpha }}}}}}}}}{*})\in {{{{{{{\mathcal{O}}}}}}}}\left(\sqrt{\frac{T\log (MT)}{N}}+\sqrt{\frac{\log ({G}_{T})}{N}}\right).$$

Thus, even if the QMLM can in principle use exponentially many parameterized gates, we can control the generalization error in terms of the number of parameterized gates used in the QMLM after optimization, and the dependence on the number of different architectures is only logarithmic. This logarithmic dependence is crucial as even in the cases when *G*_*T*_ grows exponentially with *T*, we have $\log ({G}_{T})/N\in {{{{{{{\mathcal{O}}}}}}}}(T/N)$.

### Numerical results

In this section we present generalization error results obtained by simulating the following two QML implementations: (1) using a QCNN to classify states belonging to different quantum phases, and (2) training a parameterized quantum circuit to compile a quantum Fourier transform matrix.

We begin with the quantum phase classification application. The QCNN architecture introduced in^45^ generalizes the model of (classical) convolutional neural networks with the goal of performing pattern recognition on quantum data. It is composed of so-called *convolutional* and *pooling* layers, which alternate. In a convolutional layer, a sequence of translationally invariant parameterized unitaries on neighbouring qubits is applied in parallel, which works as a filter between feature maps in different layers of the QCNN. Then, in the pooling layers, a subset of the qubits are measured to reduce the dimensionality of the state while preserving the relevant features of the data. Conditioned on the corresponding measurement outcomes, translationally invariant parameterized 1-qubit unitaries are applied. The QCNN architecture has been employed for supervised QML tasks of classification of phases of matter and to devise quantum error correction schemes^45^. Moreover, QCNNs have been shown not to exhibit barren plateaus, making them a generically trainable QML architecture^27^.

The action of a QCNN can be considered as mapping an input state *ρ*_in_ to an output state *ρ*_out_ given as ${\rho }_{{{\mbox{out}}}}={{{{{{{{\mathcal{E}}}}}}}}}_{{{{{{{{\boldsymbol{\alpha }}}}}}}}}^{{{{{{{{\rm{QCNN}}}}}}}}}({\rho }_{{{\mbox{in}}}})$. Then, given *ρ*_out_, one measures the expectation value of a task-specific Hermitian operator.

**Fig. 2:**
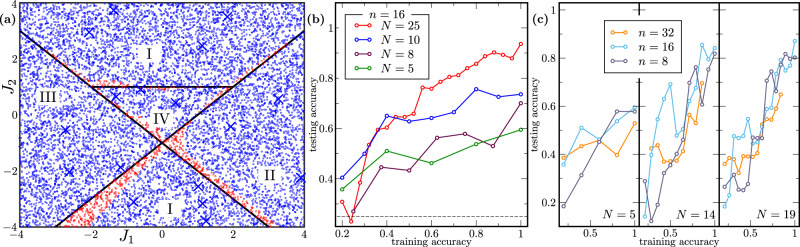
Generalization performance of quantum phase recognition. We employed the QCNN architecture for quantum phase recognition on ground states of the generalized cluster Hamiltonian *H* of Eq. (9). We evaluated the phase assigned by the QCNN to a point in the *J*_1_-*J*_2_-plane by sampling 8192 computational basis measurement outcomes and taking the least frequent outcome as the predicted phase. Panel (**a**) visualizes the performance of the QCNN for 16-qubits, trained with 30 data points, which were labelled according to the analytically determined phase diagram. Blue crosses denote training data points (not all 30 are shown). Blue (red) circles represent correctly (incorrectly) classified points. Panel (**b**) shows that, as the training data size increases, the training accuracy quickly becomes a good predictor for the testing accuracy on 10,000 randomly sampled points, i.e., the dependence of testing accuracy on training accuracy is approximately linear with slope increasing with *N*. The different points in the plot correspond to different parameter settings in the QCNN throughout the optimization. The dotted gray line shows the baseline accuracy of 25% achieved by random guessing. Panel (**c**) shows that the improvement in the slope with growing training data size is similar for different numbers of qubits, reflecting the at-worst polylogarithmic dependence of *N* on *n* predicted by our bounds.

In our implementation, we employ a QCNN to classify states belonging to different symmetry protected topological phases. Specifically, we consider the generalized cluster Hamiltonian

9 $$H=\mathop{\sum }\limits_{j=1}^{n}\left({Z}_{j}-{J}_{1}{X}_{j}{X}_{j+1}-{J}_{2}{X}_{j-1}{Z}_{j}{X}_{j+1}\right),$$

where *Z*_*i*_ (*X*_*i*_) denote the Pauli *z* (*x*) operator acting on qubit *i*, and where *J*_1_ and *J*_2_ are tunable coupling coefficients. As proved in^64^, and as schematically shown in Fig. 2, the ground-state phase diagram of the Hamiltonian of Eq. (9) has four different phases: symmetry-protected topological (I), ferromagnetic (II), anti-ferromagnetic (III), and trivial (IV). In the Methods section, we provide additional details regarding the classical simulation of the ground states of *H*.

By sampling parameters in the (*J*_1_, *J*_2_) plane, we create a training set ${\{(\left|{\psi }_{i}\right\rangle,{y}_{i})\}}_{i=1}^{N}$ composed of ground states $\left|{\psi }_{i}\right\rangle$ of *H* and their associated labels *y*_*i*_. Here, the labels are in the form of length-two bit strings, i.e., *y*_*i*_ ∈ {0, 1}^2^, where each possible bit string corresponds to a phase that $\left|{\psi }_{i}\right\rangle$ can belong to. The QCNN maps the *n*-qubit input state $\left|{\psi }_{i}\right\rangle$ to a 2-qubit output state. We think of the information about the phase as being encoded into the output state by which of the 4 computational basis effect operators is assigned the smallest probability. Namely, we define the loss function as $\ell ({{{{{{{\boldsymbol{\alpha }}}}}}}};\left|{\psi }_{i}\right\rangle,{y}_{i}):=\left\langle {y}_{i}\right|{{{{{{{{\mathcal{E}}}}}}}}}_{{{{{{{{\boldsymbol{\alpha }}}}}}}}}^{{{{{{{{\rm{QCNN}}}}}}}}}(\left|{\psi }_{i}\right\rangle \left\langle {\psi }_{i}\right|)\left|\, {y}_{i}\right\rangle$. This leads to an empirical risk given by

10 $${\hat{R}}_{S}({{{{{{{\boldsymbol{\alpha }}}}}}}})=\frac{1}{N}\mathop{\sum }\limits_{i=1}^{N}\langle\, {y}_{i}|{{{{{{{{\mathcal{E}}}}}}}}}_{{{{{{{{\boldsymbol{\alpha }}}}}}}}}^{{{{{{{{\rm{QCNN}}}}}}}}}(|{\psi }_{i} \rangle \langle {\psi }_{i}|)|{y}_{i} \rangle .$$

In Fig. 2, we visualize the phase classification performance achieved by our QCNN, trained according to this loss function, while additionally taking the number of misclassified points into account. Moreover, we show how the true risk, or rather the test accuracy as proxy for it, correlates well with the achieved training accuracy, already for small training data sizes. This is in agreement with our theoretical predictions, discussed in more detail in Supplementary Note 4, which for QCNNs gives a generalization error bound polylogarithmic in the number of qubits. We note that refs. 65, 66 observed similarly favorable training data requirements for a related task of learning phase diagrams.

Next, we turn our attention to the unitary compiling application. Compiling is the task of transforming a high-level algorithm into a low-level code that can be implemented on a device. Unitary compiling is a paradigmatic task in the NISQ era where a target unitary is compiled into a gate sequence that complies with NISQ device limitations, e.g., hardware-imposed connectivity and shallow depth to mitigate errors. Unitary compiling is crucial to the quantum computing industry, as it is essentially always performed prior to running an algorithm on a NISQ device, and various companies have their own commercial compilers^67,68^. Hence, any ability to accelerate unitary compiling could have industrial impact.

Here we consider the task of compiling the unitary *U* of the *n*-qubit Quantum Fourier Transform (QFT)^69^ into a short-depth parameterized quantum circuit *V*(***α***). For *V*(***α***) we employ the VAns (Variable Ansatz) algorithm^62,70^, which uses a machine learning protocol to iteratively grow a parameterized quantum circuit by placing and removing gates in a way that empirically leads to lower loss function values. Unlike traditional approaches that train just continuous parameters in a fixed structure circuit, VAns also trains discrete parameters, e.g., gate placement or type of gate, to explore the architecture hyperspace. In Supplementary Note 5, we apply our theoretical results in this compiling scenario.

The training set for compilation is of the form ${\{\left|{\psi }_{i}\right\rangle,U\left|{\psi }_{i}\right\rangle \}}_{i=1}^{N}$, consisting of input states $\left|{\psi }_{i}\right\rangle$ and output states obtained through the action of *U*. The $\left|{\psi }_{i}\right\rangle$ are drawn independently from an underlying data-generating distribution. In our numerics, we consider three such distributions: (1) random computational basis states, (2) random (non-orthogonal) low-entangled states, and (3) Haar random *n*-qubit states. Note that states in the first two distributions are easy to prepare on a quantum computer, whereas states from the last distribution become costly to prepare as *n* grows. As the goal is to train *V*(***α***) to match the action of *U* on the training set, we define the loss function as the squared trace distance between $U\left|{\psi }_{i}\right\rangle$ and $V({{{{{{{\boldsymbol{\alpha }}}}}}}})\left|{\psi }_{i}\right\rangle$, i.e., $\ell (\alpha ;\left|{\psi }_{i}\right\rangle,U\left|{\psi }_{i}\right\rangle ):=||U\left|{\psi }_{i}\right\rangle \left\langle {\psi }_{i}\right|{U}^{{{{\dagger}}} }-V({{{{{{{\boldsymbol{\alpha }}}}}}}})\left|{\psi }_{i}\right\rangle \left\langle {\psi }_{i}\right|V{({{{{{{{\boldsymbol{\alpha }}}}}}}})}^{{{{\dagger}}} }|{|}_{1}^{2}$. This leads to the empirical risk

11 $${\hat{R}}_{S}({{{{{{{\boldsymbol{\alpha }}}}}}}})=\frac{1}{N}\mathop{\sum }\limits_{i=1}^{N}||U\left|{\psi }_{i}\right\rangle \left\langle {\psi }_{i}\right|{U}^{{{{\dagger}}} }-V({{{{{{{\boldsymbol{\alpha }}}}}}}})\left|{\psi }_{i}\right\rangle \left\langle {\psi }_{i}\right|V{({{{{{{{\boldsymbol{\alpha }}}}}}}})}^{{{{\dagger}}} }|{|}_{1}^{2},$$

where ∣∣ ⋅ ∣∣_1_ indicates the trace norm.

**Fig. 3:**
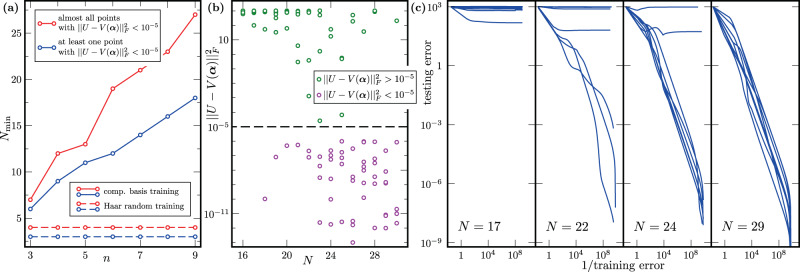
Generalization performance of variational unitary compiling. We employed a variable structure QMLM (as discussed near Theorem 4). Panel (**a**) shows the dependence of ${N}_{\min }$, the minimum training data size for accurate compilation, on *n*, the number of qubits. Accurate compilation is defined as achieving $\parallel U-V({{{{{{{\boldsymbol{\alpha }}}}}}}}){\parallel }_{F}^{2} \, < \, 1{0}^{-5}$ on 1 out of 8 (blue) or on 7 out of 8 (red) runs. For training data with random computational basis inputs (solid lines), ${N}_{\min }$ scales linearly in *n*. When training on examples with Haar random inputs (dashed lines), ${N}_{\min }$ is constant up to system size *n* = 9. In Panel (**b**), for *n* = 9 qubits, we plot the prediction error of successfully trained (training cost < 10^−8^) runs for 8 training data sets with *N* = 16 to *N* = 30 random computational basis inputs. Panel (**c**) shows the dependence of the testing error on the reciprocal of the training error for different training data sizes, in the case of 9 qubits. Here, the data consisted of random computational basis states and the corresponding outputs. As *N* increases, small training error becomes a more reliable predictor for small testing error. Each subplot shows 8 different training runs, trained on different training data sets.

Figure 3 shows our numerical results. As predicted by our analytical results, we can, with high success probability, accurately compile the QFT when training on a data set of size polynomial in the number of qubits. Our numerical investigation shows a linear scaling of the training requirements when training on random computational basis states. This better than the quadratic scaling implied by a direct application of our theory, which holds for any arbitrary data-generating distribution. Approximate implementations of QFT with a reduced number of gates^71^, combined with our results, could help to further study this apparent gap theoretically. When training on Haar random states, our numerics suggest that an even smaller number of training data points is sufficient for good generalization: Up to *n* = 9 qubits, we generalize well from a constant number of training data points, independent of the system size.

**Fig. 4:**
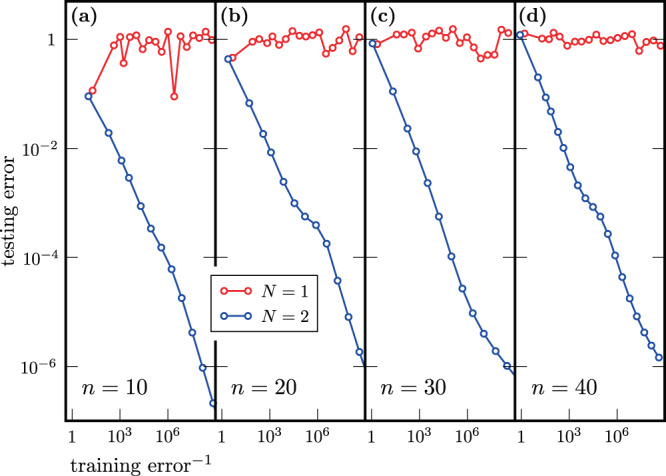
Performance of variational unitary compiling when initializing near the solution. Each panel shows the results of a single randomly initialized training run, where we used randomly drawn low-entangled states for training. The testing error on 20 test states, which we allow to be more strongly entangled than the states used during training, is plotted versus the reciprocal of the training error for training data sizes *N* = 1, 2, for different system sizes *n*. A training data set of size *N* = 1 is not sufficient to guarantee good generalization: Even with decreasing training error, the testing error remains large. In contrast, assuming favorably initialized training, *N* = 2 training data points suffice for good generalization, even for up to *n* = 40 qubits.

Even more striking are our results when initializing close to the solution. In this case, as shown in Fig. 4, we find that two training data points suffice to obtain accurate generalization, which holds even up to a problem size of 40 qubits. Our theoretical results in Theorem 3 do predict reduced training requirements when initializing near the solution. Hence, the numerics are in agreement with the theory, although they paint an even more optimistic picture and suggest that further investigation is needed to understand why the training data requirements are so low. While the assumption of initialization near the solution is only viable assuming additional prior knowledge, it could be justified in certain scenarios. For example, if the unitaries to be compiled depend on a parameter, e.g., time, and if we have already compiled the unitary for one parameter setting, we might use this as initialization for unitaries with a similar parameter.

## Discussion

We conclude by discussing the impact of our work on specific applications, a comparison to prior work, the interpretation of our results from the perspective of quantum advantage, and some open questions.

We begin with a discussion of the impact on specific applications. Quantum phase classification is an exciting application of QML, to which Ref. 45 has successfully applied QCNNs. However, Ref. 45 only provided a heuristic explanation for the good generalization performance of QCNNs. Here, we have presented a rigorous theory that encompasses QCNNs and explains their performance, and we have confirmed it numerically for a fairly complicated phase diagram and a wide range of system sizes. In particular, our analysis allows us to go beyond the specific model of QCNNs and extract general principles for how to ensure good generalization. As generating training data for this problem asks an experimenter to prepare a variety of states from different phases of matter, which will require careful tuning of different parameters in the underlying Hamiltonian, good generalization guarantees for small training data sizes are crucial to allow for the implementation of phase classification through QML in actual physical experiments.

Several successful protocols for unitary compiling make use of training data^52–54^. However, prior work has relied on training data sets whose size scaled exponentially with the number of qubits. This scaling is problematic, both because it suggests a similarly bad scaling of the computational complexity of processing the data and because generating training data can be expensive in actual physical implementations. Our generalization bounds provide theoretical guarantees on the performance that unitary compiling with only polynomial-size training data can achieve, for the relevant case of efficiently implementable target unitaries. As we have numerically demonstrated in the case of the Quantum Fourier Transform, this significant reduction in training data size makes unitary compiling scalable beyond what previous approaches could achieve. Moreover, our results provide new insight into why the VAns algorithm^62^ is successful for unitary compiling. We believe that the QML perspective on unitary compiling advocated for in this work will lead to new and improved ansätze, which could scale to even larger systems.

Recent methods for variational dynamical simulation rely on quantum compiling to compile a Trotterized unitary into a structured ansatz with the form of a diagonalization^48,49,72,73^. This technique allows for quantum simulations of times longer than an iterated Trotterization because parameters in the diagonalization may be changed by hand to provide longer-time simulations with a fixed depth circuit. We expect the quantum compiling results presented here to carry over to this application. This will allow these variational quantum simulation methods to use fewer training resources (either input-output pairs, or entangling auxiliary systems), yet still achieve good generalization and scalability.

Discovering quantum error correcting codes can be viewed as an optimization problem^44,45,74–78^. Furthermore, it can be thought of as a machine learning problem, since computing the average fidelity of the code involves training data (e.g., chosen from a 2-design^44^). Significant effort has been made to solve this problem on classical computers^74–78^. Such approaches can benefit from our generalization bounds, potentially leading to faster classical discovery of quantum codes. More recently, it was proposed to use near-term quantum computers to find such codes^44,45^. Again our bounds imply good generalization performance with small training data for this application, especially for QCNNs^45^, due to their logarithmic number of parameters.

Finally, autoencoders and generative adversarial networks (GANs) have recently been generalized to the quantum setting^13,43,79,80^. Both employ training data, and hence our generalization bounds provide quantitative guidance for how much training data to employ in these applications. Moreover, our results can provide guidance for ansatz design. While there is no standard ansatz yet for quantum autoencoders or quantum GANs, ansätze with a minimal number of parameters will likely lead to the best generalization performance.

Next, we give a comparison to previously known results. Some prior works have studied the generalization capabilities of quantum models, among them the classical learning-theoretic approaches of^81–89^; the more geometric perspective of^17,18^; and the information-theoretic technique of^20,37^. Independently of this work, Ref. 38 also investigated covering numbers in QMLMs. However our bounds are stronger, significantly more general, and broader in scope. We give a detailed comparison of our results to related work in Supplementary Note 1.

To view our results in the context of the quest for quantum advantage, it is important to note that we do not prove a quantum advantage of quantum over classical machine learning. However, generalization bounds for QMLMs are necessary to understand their potential for quantum advantage. Namely, QMLMs can outperform classical methods, assuming both achieve small training error, only in scenarios in which QMLMs generalize well, but classical ML methods do not. We therefore consider our results a guide in the search for quantum advantage of QML: We need to identify a task in which QMLMs with few trainable gates achieve small training error, but classical models need substantially higher model complexity to achieve the same goal. Then, our bounds guarantee that the QMLM performs well also on unseen data, but we expect the classical model to generalize poorly due to the high model complexity.

We conclude with some open questions. For QMLMs with exponentially many independently trainable gates, our generalization error bounds scale exponentially with *n*, and hence we do not make non-trivial claims about this regime. However, this does not yet imply that exponential-size QMLMs have bad generalization behavior. Whether and under which circumstances this is indeed the case is an interesting open question (e.g., see^17,37^ for some initial results). More generally, one can ask: Under what circumstances will a QMLM, even one of polynomial size, outperform our general bound. For example, if we have further prior knowledge about the loss, arising from specific target applications, it might be possible to use this information to tighten our generalization bounds. Moreover, as our generalization bounds are valid for arbitrary data-generating distributions, they may be overly pessimistic for favorable distributions. Concretely, in our numerical experiments for unitary compiling, highly entangled states were more favorable than especially efficiently preparable states from the perspective of generalization. It may thus be interesting to investigate distribution-specific tightenings of our results. Finally, it may be fruitful to combine the generalization bounds for QMLMs studied in this work and the effect of data encodings in^86^ to yield a better picture on generalization in quantum machine learning.

## Methods

This section gives an overview over our techniques. First, we outline the proof strategy that leads to the different generalization bounds stated above. Second, we present more details about our numerical investigations.

### Analytical methods

An established approach to generalization bounds in classical statistical learning theory is to bound a complexity measure for the class under consideration. Metric entropies, i.e., logarithms of covering numbers, quantify complexity in exactly the way needed for generalization bounds, as one can show using the chaining technique from the theory of random processes^90,91^. Therefore, a high level view of our proof strategy is: We establish novel metric entropy bounds for QMLMs and then combine these with known generalization results from classical learning theory. The strongest form of our generalization bounds is the following.

#### Theorem 5

(*Mother theorem*). Consider a QMLM with an arbitrary number of parameterized local quantum channels, where for each *T* > 0, we have *G*_*T*_ different QMLM architectures with *T* trainable local gates. Suppose that after optimizing on the training data, the QMLM has *T* independently parameterized local quantum channels, where the *t*^th^ channel is reused at most *M* times and is changed by Δ_*t*_ during the optimization. Without loss of generality, assume Δ_1_≥…≥Δ_*T*_. Then with high probability over input training data of size *N*, we have

12 $${{{{{{\mathrm{gen}}}}}}}\,({{{{{{{{\boldsymbol{\alpha }}}}}}}}}{*})\in {{{{{{{\mathcal{O}}}}}}}}\left(\mathop{\min }\limits_{K=0,\ldots,T}\, f(K)+\sqrt{\frac{\log ({G}_{T})}{N}}\right),$$

where $$f(K):=\sqrt{\frac{K\log (MT)}{N}}+\mathop{\sum }\limits_{k=K+1}^{T}M{{{\Delta }}}_{k}$$.

We give a detailed proof in Supplementary Note 3. There, we also describe a variant in case the loss function cannot be evaluated exactly, but only estimated statistically. Here, we present only a sketch of how to prove Theorem 5.

Before the proof sketch, however, we discuss how Theorem 5 relates to the generalization bounds stated above. In particular, we demonstrate how to obtain Theorems 1, 2, 3, and 4 as special cases of Theorem 5.

In the scenario of Theorem 1, the QMLM architecture is fixed in advance, each trainable map is only used once, and we do not take properties of the optimization procedure into account. In the language of Theorem 5, this means: There exists a single *T* > 0 with *G*_*T*_ = 1 and we have ${G}_{\tilde{T}}=0$ for all $\tilde{T}\ne T$. Also, *M* = 1. And instead of taking the minimum over *K* = 1, …, *T*, we consider the bound for *K* = *T*. Plugging this into the generalization bound of Theorem 5, we recover Theorem 1.

Similarly, Theorem 5 implies Theorems 2, 3, and 4. Namely, if we take *G*_*T*_ = 1 and ${G}_{\tilde{T}}=0$ for all $\tilde{T}\ne T$, and evaluate the bound for *K* = *T*, we recover Theorem 2. Choosing *G*_*T*_ = 1 and ${G}_{\tilde{T}}=0$ for all $\tilde{T}\ne T$, the bound of Theorem 5 becomes that of Theorem 3. Finally, we can obtain Theorem 4 by bounding the minimum in Theorem 5 in terms of the expression evaluated at *K* = *T*.

Now that we have established that Theorem 5 indeed implies generalization bounds for all the different scenarios depicted in Fig. 1, we outline its proof. The first central ingredient to our reasoning are metric entropy bounds for the class of all *n*-qubit CPTP maps that a QMLM as described in Theorem 5 can implement, where the distance between such maps is measured in terms of the diamond norm. Note: The trivial metric entropy bound obtained by considering this class of maps as compact subset of an Euclidean space of dimension exponential in *n* is not sufficient for our purposes since it scales exponentially in *n*. Instead, we exploit the layer structure of QMLMs to obtain a better bound. More precisely, we show: If we fix a QMLM architecture with *T* trainable 2-qubit maps and a number of maps 0 ⩽ *K* ⩽ *T*, and we assume (data-dependent) optimization distances Δ_1_⩾…⩾Δ_*T*_, then it suffices to take (*ε*/*KM*)-covering nets for each of the sets of admissible 2-qubit CPTP maps for the first *K* trainable maps to obtain a $(\varepsilon+\mathop{\sum }\nolimits_{k=K+1}^{T}M{{{\Delta }}}_{k})$-covering net for the whole QMLM. The cardinality of a covering net built in this way, crucially, is independent of *n*, but depends instead on *K*, *M*, and *T*. In detail, its logarithm can effectively be bounded as $\in {{{{{{{\mathcal{O}}}}}}}}\left(K\log \left(MT/\varepsilon \right)\right)$. This argument directly extends from the 2-local to the κ-local case, as we describe in Supplementary Note 3.

Now we employ the second core ingredient of our proof strategy. Namely, we combine a known upper bound on the generalization error in terms of the expected supremum of a certain random process with the so-called chaining technique. This leads to a generalization error bound in terms of a metric entropy integral. As we need a non-standard version of this bound, we provide a complete derivation for this strengthened form. This then tells us that, for each fixed *T*, *M*, *K*, and Δ_1_⩾…⩾Δ_*T*_, using the covering net constructed above, we can bound the generalization error as ${{{{{{\mathrm{gen}}}}}}}\,({{{{{{{{\boldsymbol{\alpha }}}}}}}}}{*})\in {{{{{{{\mathcal{O}}}}}}}}(\sqrt{K\log (MT)/N}+\mathop{\sum }\nolimits_{k=K+1}^{T}M{{{\Delta }}}_{k})$, with high probability.

The last step of the proof consists of two applications of the union bound. The first instance is a union bound over the possible values of *K*. This leads to a generalization error bound in which we minimize over *K* = 0, …, *T*. So far, however, the bound still applies only to any QMLM with fixed architecture. We extend it to variable QMLM architectures by taking a second union bound over all admissible numbers of trainable gates *T* and the corresponding *G*_*T*_ architectures. As this is, in general, a union bound over countably many events, we have to ensure that the corresponding failure probabilities are summable. Thus, we invoke our fixed-architecture generalization error bound for a success probability that is proportional to ${({G}_{T}{T}^{2})}^{-1}$. In that way, the union bound over all possible architectures yields the logarithmic dependence on *G*_*T*_ in the final bound and completes the proof of Theorem 5.

### Numerical methods

This section discusses numerical methods used throughout the paper. The subsections give details on computational techniques applied to phase classification of the cluster Hamiltonian in Eq. (9) and Quantum Fourier Transform compilation.

#### Phase classification

The training and testing sets consist of ground states $\left|{\psi }_{i}\right\rangle$ of the cluster Hamiltonian in Eq. (9), computed for different coupling strengths (*J*_1_, *J*_2_). The states $\left|{\psi }_{i}\right\rangle$ were obtained with the translation invariant Density Matrix Renormalization Group^92^. The states in the training set (represented by blue crosses in Fig. 2a) are chosen to be away from phase transition lines, so accurate description of the ground states is already achieved at small bond dimension *χ*. That value determines the cost of further computation involving the states $\left|{\psi }_{i}\right\rangle$ and we keep it small for efficient simulation.

We use Matrix Product State techniques^93^ to compute and optimize the empirical risk in Eq. (10). The main part of that calculation is the simulation of the action of the QCNN ${{{{{{{{\mathcal{E}}}}}}}}}_{{{{{{{{\boldsymbol{\alpha }}}}}}}}}^{{{{{{{{\rm{QCNN}}}}}}}}}$ on a given ground state $\left|{\psi }_{i}\right\rangle$. The map ${{{{{{{{\mathcal{E}}}}}}}}}_{{{{{{{{\boldsymbol{\alpha }}}}}}}}}^{{{{{{{{\rm{QCNN}}}}}}}}}$ consists of alternating convolutional and pooling layers. In our implementation the layers are translationally invariant and are represented by parameterized two-qubit gates. The action of a convolutional layer on an MPS amounts to updating two nearest neighbor MPS tensors in a way similar to the time-evolving block decimation algorithm^94^. The pooling layer is simulated in two steps. First, we simulate the action of all two-qubit gates on an MPS. This is analogous to the action of a convolutional layer, but performed on a different pair of nearest neighbor MPS tensors. This step is followed by a measurement of half of the qubits. We use the fact that the MPS can be written as a unitary tensor network and hence allows for perfect sampling techniques^95^. The measurement step results in a reduction of the system size by a factor of two.

We repeat the application of convolutional and pooling layers using the MPS as described above until the system size becomes small enough to allow for an exact description. A few final layers are simulated in a standard way and the empirical risk is given by a two-qubit measurement according to the label *y*_*i*_, as in Eq. (10). The empirical risk is optimized with the Simultaneous Perturbation Stochastic Approximation algorithm^96^. We grow the number of shots used in pooling layer measurements as the empirical risk is minimized. This results in a shot-frugal optimization^97^, as one can control the accuracy of the gradient based on the current optimization landscape.

#### Unitary compiling

In the Numerical results section, we show that the task of unitary compilation can be translated into minimization of the empirical risk ${\hat{R}}_{S}({{{{{{{\boldsymbol{\alpha }}}}}}}})$ defined in Eq. (11). Here, ***α*** = (***θ***, ***k***) denotes a set of parameters that specifies a trainable unitary *V*(***α***). The optimization is performed in the space of all shallow circuits. It has discrete and continuous components. The discrete parameters ***k*** control the circuit layout, that is, the placement of all gates used in the circuit. Those gates are described by the continuous parameters ***θ***. The optimization $\mathop{\min }\limits_{{{{{{{{\boldsymbol{\alpha }}}}}}}}}{\hat{R}}_{S}({{{{{{{\boldsymbol{\alpha }}}}}}}})$ is performed with the recently introduced VAns algorithm^62,70^. The unitary *V*(***α***) is initialized with a circuit that consists of a few randomly placed gates. In subsequent iterations, VAns modifies the structure parameter ***k*** according to certain rules that involve randomly placing a resolution of the identity and removing gates that do not significantly contribute to the minimization of the empirical risk ${\hat{R}}_{S}({{{{{{{\boldsymbol{\alpha }}}}}}}})$. The qFactor algorithm^54^, modified to work with a set of pairs of states as opposed to a target unitary, is used to optimize over continuous parameters ***θ*** for fixed ***k***. This optimization is performed after each update to the structure parameter ***k***. In subsequent iterations, VAns makes a probabilistic decision whether the new set of parameters ${{{{{{{\boldsymbol{{\alpha }}}}}}}^{\prime}}}$ is kept or rejected. This decision is based on the change in empirical risk ${\hat{R}}_{S}({{{{{{{\boldsymbol{{\alpha }}}}}}}^{\prime}}})-{\hat{R}}_{S}({{{{{{{\boldsymbol{\alpha }}}}}}}})$, an artificial temperature *T*, and a factor Λ that sets the penalty for growing the circuit too quickly. To that end, we employ a simulated annealing technique, gradually decreasing *T* and Λ, and repeat the iterations described above until ${\hat{R}}_{S}({{{{{{{\boldsymbol{\alpha }}}}}}}})$ reaches a sufficiently small value.

Let us now discuss the methods used to optimize the empirical risk when *V*(***α***) is initialized close to the solution. Here, we start with a textbook circuit for performing the QFT and modify it in the following way. First, the circuit is rewritten such that it consists of two-qubit gates only. Next, each two-qubit gate *u* is replaced with $u^{\prime}=u{e}^{i\delta h}$, where *h* is a random Hermitian matrix and *δ* is chosen such that $||u-u^{\prime}||=\epsilon$ for an initially specified *ϵ*. The results presented in the Numerical results section are obtained with *ϵ* = 0.1. The perturbation considered here does not affect the circuit layout and hence the optimization over continuous parameters ***θ*** is sufficient to minimize the empirical risk ${\hat{R}}_{S}({{{{{{{\boldsymbol{\alpha }}}}}}}})$. We use qFactor to perform that optimization.

The input states $\left|{\psi }_{i}\right\rangle$ in the training set ${\{\left|{\psi }_{i}\right\rangle,{U}_{{{{{{{{\rm{QFT}}}}}}}}}\left|{\psi }_{i}\right\rangle \}}_{i=1}^{N}$ are random MPSs of bond dimension *χ* = 2. The QFT is efficiently simulable^98^ for such input states, which means that ${U}_{{{{{{{{\rm{QFT}}}}}}}}}\left|{\psi }_{i}\right\rangle$ admits an efficient MPS description. Indeed, we find that a bond dimension *χ* < 20 is sufficient to accurately describe ${U}_{{{{{{{{\rm{QFT}}}}}}}}}\left|{\psi }_{i}\right\rangle$. In summary, the use of MPS techniques allows us to construct the training set efficiently. Note that the states $V({{{{{{{\boldsymbol{\alpha }}}}}}}})\left|{\psi }_{i}\right\rangle$ are in general more entangled than ${U}_{{{{{{{{\rm{QFT}}}}}}}}}\left|{\psi }_{i}\right\rangle$, especially at the beginning of the optimization. Because of that, we truncate the evolved MPS during the optimization. We find that a maximal allowed bond dimension of 100 is large enough to perform stable, successful minimization of the empirical risk with qFactor. The testing is performed with 20 randomly chosen initial states. We test with bond dimension *χ* = 10 MPSs, so the testing is done with more strongly entangled states than the training. Additionally, for system sizes up to 16 qubits, we verify that the trained unitary *V* is close (in the trace norm) to *U*_QFT_, when training is performed with at least two states.

## Supplementary information

Supplementary Information

## Data Availability

The data generated and analyzed during the current study are available from the authors upon request.

## Code availability

Further implementation details are available from the authors upon request.
